# Physiology of Astroglial Excitability

**DOI:** 10.1093/function/zqaa016

**Published:** 2020-09-04

**Authors:** Alexei Verkhratsky, Alexey Semyanov, Robert Zorec

**Affiliations:** 1 Faculty of Biology, Medicine and Health, The University of Manchester, Manchester, M13 9PT, UK; 2 Achucarro Center for Neuroscience, Ikerbasque, 48011 Bilbao, Spain; 3 Shemyakin-Ovchinnikov Institute of Bioorganic Chemistry, Russian Academy of Sciences, Moscow 117997, Russia; 4 Faculty of Biology, Moscow State University, Moscow, Russia; 5 Sechenov First Moscow State Medical University, Moscow, Russia; 6 Celica Biomedical, Ljubljana 1000, Slovenia; 7 Laboratory of Neuroendocrinology-Molecular Cell Physiology, Institute of Pathophysiology, Faculty of Medicine, University of Ljubljana, Ljubljana 1000, Slovenia

**Keywords:** astrocyte, astrocytic processes, calcium signaling, sodium signaling, ionic signaling, astroglial excitability

## Abstract

Classic physiology divides all neural cells into excitable neurons and nonexcitable neuroglia. Neuroglial cells, chiefly responsible for homeostasis and defense of the nervous tissue, coordinate their complex homeostatic responses with neuronal activity. This coordination reflects a specific form of glial excitability mediated by complex changes in intracellular concentration of ions and second messengers organized in both space and time. Astrocytes are equipped with multiple molecular cascades, which are central for regulating homeostasis of neurotransmitters, ionostasis, synaptic connectivity, and metabolic support of the central nervous system. Astrocytes are further provisioned with multiple receptors for neurotransmitters and neurohormones, which upon activation trigger intracellular signals mediated by Ca^2+^, Na^+^, and cyclic AMP. Calcium signals have distinct organization and underlying mechanisms in different astrocytic compartments thus allowing complex spatiotemporal signaling. Signals mediated by fluctuations in cytosolic Na^+^ are instrumental for coordination of Na^+^ dependent astrocytic transporters with tissue state and homeostatic demands. Astroglial ionic excitability may also involve K^+^, H^+,^ and Cl^−^. The cyclic AMP signalling system is, in comparison to ions, much slower in targeting astroglial effector mechanisms. This evidence review summarizes the concept of astroglial intracellular excitability.

## The Concept of Excitability

The concept of physiological excitability and the definition of excitable and nonexcitable tissues was formulated by Albrecht von Haller, who exposed different organs or their parts to injury, by squeezing and stinging, by sprinkling with cold, hot, or corrosive substances or by electrocuting. Analyzing responses to such interrogations, von Haller proposed to classify all organs into sensible (*sensibilis*) and irritable (*irritabilis*) ones.^1^ In addition to the tissues that actively responded to various manipulations, von Haller also noticed a third type of organs and tissues, which were neither sensible nor irritable; he named this type of tissue the *Zellgewebsfaser* or cell tissue fiber that came together to form the *Zellgewebe* (“cellular tissue”). This was an inert tissue, forming a filling or basic substance that surrounds and covers all components of the organism being in a way a predecessor of the connective tissue of Rudolf Virchow.

Although the cellular theory had not yet been established by the time of von Haller’s work the notion of the cell as an elementary living entity has been considered; the term being invented by Robert Hook in the 1650s.^2^ The first description of brain cells was made by Marcello Malpighi who described the cortical tissue as being formed from many globules or “little glands”^3^; similar structures were also observed by Antonie van Leeuwenhoek.^4^ The first detailed description of brain elementary structures were made by Emanuel Swedenborg in 1740s, who envisaged the nervous tissue as made from functionally independent globules or *cerebellulas* (minute brains) that are connected by nerve fibers, which receive sensations from or project motor impulses into the peripheral organs. Swedenborg described these structures as the substrates for brain function. He wrote “From each cortical gland proceeds a single nerve fiber; this is carried down into the body, in order that it may take hold of some part of a sensation or produce some action.”^5^ The first documented drawing of nerve cells (known as “*globules*” or “*kugeln*” both denoting spheres) from the microscopic observations were made by Christian Gottfried Ehrenberg^6^ and Jan Evangelista Purkinje^7^; while the term “nerve cell” was introduced by Robert Bently Todd^8^ in 1845.

A pupil and assistant of Purkinje, Gabriel Gustav Valentin was the first to contemplate two types of nervous elements, which he called nervous masses. One of these masses representing “the creative, active, higher principle” while the second “the receiving and guiding, passive, lower principle.”^9^ The active substance was represented by spherical elements (*Kugeln der Belegungsmassen*) and nerve fibers (*Primitivfasern*), whereas the passive substance was defined as intermediate substance (*Zellgewebescheide*) made from fibers and threads. The ideas about specific brain connective tissue were further developed by Carl von Rokitansky,^10^ and Rudolf Virchow who, in 1856 defined the connective tissue of the brain as “…connective substance forms in the brain, the spinal cord and the higher sensory nerves a type of putty (neuroglia), in which the nervous elements are embedded.”^11^ Although Virchow most likely considered neuroglia as an acellular *bona fide* connective tissue, glial cells have been visualized and identified by many neuroanatomists^12^ and their roles have been considered by physiologists. Many active contributions of neuroglia to numerous brain functions have been proposed, these range from interfacing the vasculature with brain parenchyma, thereby regulating local hyperemia, to control over synaptic transmission and brain states, such as the sleep-wake transition.^^13–16^^ These considerations changed the role of glia from being regarded as simply a connective tissue to an active counterpart of neurons in executing brain functions.

The advent of electrophysiology and intracellular recordings led to a detailed characterization of electrical excitability of nerves, muscle, and neurons. The very first observation of experimentally evoked muscle contractions were made in the 1660s by Jan Swammerdam, who designed the classic frog neuromuscular preparation.^17^^,^^18^ The discovery of animal electricity was made in 1780–1790s by Luigi Galvani working together with his wife Lucia Galeazzi and his nephew Giovanni Aldini. Galvani recorded electrical excitation of the nerve–muscle preparation, described the relationship between stimulus intensity and muscle contraction, defined the refractory period, and above all demonstrated the propagating wave of excitation through nerve and muscle, known to us as the action potential.^19^^,^^20^

Some 150 years later, the seminal discoveries of Hodgkin and Huxley provided the first quantitative description of the ionic conductance changes underlying the action potential,^21^ while the emergence of patch-clamp techniques^22^ and molecular cloning^23^ identified structural and functional properties of ion channels and established mechanisms underlying electrical excitability of neurons. The first electrophysiological recordings from glial cells *in vivo*, in organotypic cultures, in isolated optic nerve preparations from amphibians or in the isolated ganglionic chain of the leech^24–27^ revealed the passive properties of the membranes of these cells as well as the inability of glia to generate action potentials. These experiments also found that glial cells respond with small (several mV) depolarizations to neuronal activity or to some neurotransmitters; all these responses were attributed to originate from K^+^ accumulation in the extracellular space.

When the technique for making purified glial cell cultures was developed^28^ and these cultured cells were interrogated with microelectrodes and patch-clamp approaches the functional expression of neurotransmitter receptors was discovered.^29^^,^^30^ Subsequent experiments found that glial cells are capable of expressing virtually every type of neurotransmitter receptor in existence, and moreover *in vivo* expression of these receptors was tightly regulated by the neurochemical environment: the neuroglial receptor pattern is tailored to neurotransmitters operating in a particular brain region.^31^ When cultured neuroglial cells were probed with Ca^2+^indicators, it turned out that chemical or mechanical stimulation of glia almost invariably triggered complex cytoplasmic Ca^2+^ signals, which, in a form of Ca^2+^ waves, could propagate over long distances through the gap-junction connected glial syncytium.^^32–35^^ Thus, the concept of calcium excitability of neuroglia was developed.^36^ Ensuing years brought further advancement in the understanding of astroglial excitability, as it turned out that stimulation of astrocytes is associated with substantial Na^+^ fluxes that generate cytoplasmic Na^+^ signals as well as with highly organized changes in cytosolic second messengers such as InsP_3_ and cAMP; the former being linked to Ca^2+^ signaling whereas the latter being connected with numerous intracellular enzymatic cascades and influenced by Ca^2+^. Consequently, a coherent concept of intracellular astroglial excitability is in need of definition.

## Astroglial Intracellular Excitability

Appearance of the central nervous system (CNS), which emerged early in evolution, was accompanied by division of neural cells into neuron, which represent executive arm responsible for sensory input, information processing, and initiation of peripheral responses and homeostatic and defensive neuroglia. Neuroglial cells of the brain and the spinal cord are classified into macroglia (the cells of neuroepithelial origin further subdivided into astrocytes and oligodendroglia) and microglia, which are scions of fetal macrophages invading the CNS early in the development. Neurons are universally considered as the only excitable cells of the nervous system; they generate fast action potentials, which are conducted over large distances and initiate neurotransmitter release responsible for synaptic connectivity. Nonetheless, fast signaling (in addition to relatively slow ones) does also occur in glial cells, which respond to physiological stimulation with transient fluctuations in their ionic content; these ionic signals are the substrate for rapid stimulus-induced glial excitability.

Astrocytes are the principal homeostatic cells of the CNS, which constantly adapt operation of elaborated homeostatic molecular cascades to neuronal activity and brain state. Astrocytes control CNS homeostasis at many levels. First and foremost astrocytes are responsible for CNS ionostasis—the ionic composition of the interstitial fluid, which are tightly associated with changes in brain state, such as sleep and arousal.^37^ Astroglial cells are fundamental for uptake and catabolism of the principal neurotransmitters including glutamate, noradrenaline (NA), GABA, glycine, and adenosine^38–42^; astrocytes also supply neurons with neurotransmitter precursors such as glutamine or l-serine.^43^^,^^44^ Astrocytes provide neurons with energy substrates^45^ and contribute to regulation of capillary blood flow and local functional hyperemia.^46^^,^^47^ They also provide for water transport from the perivascular space thus supporting the operation of glymphatic system,^48^ astrocytes sustain the blood–brain barrier,^49^ and participate in the defense of the CNS through mounting reactive astrogliosis.^50^ Furthermore, astrocytes act as baroreceptors to sense cerebral perfusion and control systemic circulation.^51^ All these functions and processes need to be coordinated with neural activity, which stipulates the existence of sophisticated signaling underlying astroglial activation in various physiological and pathological contexts; this activation is the result of astroglial excitability.

Sensing the neural tissue environment involves the stimulation of astroglial membrane receptors. Activation of these receptors does not trigger regenerative transmembrane depolarization, instead it produces changes in intracellular ion activity reflecting changes in free ion concentration ([ion]_i_), which regulate astroglial physiological activity. Similarly to neurons, astrocytes, are activated in response to sensory stimulation; numerous experiments *in vivo* in anesthetized and awake animals have demonstrated synchronous cytosolic [Ca^2+^]_i_ transients engulfing groups of astrocytes in the sensory cortex.^52^^,^^53^ Synaptic transmission is similarly associated with activation of astrocytes: synaptically released glutamate induces local astroglial Ca^2+^ signals originating from endoplasmic reticulum (ER) Ca^2+^ release and/or from Ca^2+^ entry across the plasmalemma^54^^,^^55^; at the same time glutamate is taken up into astrocytes by Na^+^-dependent transporters, generating a massive Na^+^ influx, which triggers cytosolic Na^+^ signals.^56^^,^^57^ These ionic signals in turn affect various intracellular sensors, which regulate astroglial homeostasis pathways and astroglial morphological plasticity.^58^^,^^59^

Changes in the state of the brain—arousal, stress, concentration, behavior - are associated with activation of locus coeruleus (LC), which represents the prime neuronal plexus localized in the brain stem; projections of the LC neurons synchronously release NA in various brain and spinal cord regions. In the adult human brain, the LC consists of only around 50 000 neurons^60^; these neurons deliver ∼70% of all NA in the CNS.^61^ The hallmark of LC-mediated activities include arousal, attention, memory formation, sleep regulation, emotional balance, and cognitive control, all depending on NA-mediated morphologic neuroplasticity and metabolic support.^62^^,^^63^

Astrocytes are major targets of NA in the CNS; mature astrocytes express adrenoceptors of both α and β varieties while the density of adrenoceptors in astrocytic processes seems to be significantly higher than in neurons.^64^ The action of NA on astroglia results in the activation of fast ionic signals and much slower stimulus-response signaling associated with changes in the concentration of the second messenger 3′,5′-cyclic adenosine monophosphate (cAMP), triggering downstream enzymatic cascades, which regulate numerous processes, including the control of gene transcription, needed for astroglial plasticity during learning and memory.^65^

## Ionic Excitability of Astroglia

Maintenance of cellular ionic homeostasis is one of the most fundamental conditions for life; all living organisms on planet Earth are keeping the ionic composition of cytosol and organelles under tight control at the expense of considerable energy. Ionic gradients between the extracellular space and the cytosol are driving ion fluxes. These ionic fluxes originate from opening of ion channels following for example an environmental stress in unicellular organisms or due to release of chemical messengers in multicellular ones. Thus, from the very beginning of life, ions evolved as dynamic intracellular signalers coupling extrinsic challenges to intracellular processes. Conceptually, living cells are constantly balancing the preservation of their ionic composition with generation of ionic fluctuations organized in space and time. To achieve this steady-state, evolution selected transport systems moving ions along and against concentration gradients. In essence, all changes in the cytosolic concentration of any ion, di- or monovalent, can regulate/modulate various cellular events, and hence may act as second messengers in biological systems. Ionic signalingis shaped by dynamic interactions of diffusion (ion movement along an electrochemical gradient) and primary or secondary ion transport (often against electrochemical gradients), which requires energy. All these molecular cascades are in operation in astrocytes.

### Astroglial Calcium Signaling

It is universally acknowledged that an increase in the Ca^2+^ concentration acts as a ubiquitous physiological signal, operating in most (if not in all) cells and tissues. Changes in the Ca^2+^ concentration in various cellular compartments trigger or regulate a wide variety of cellular processes. The Ca^2+^ homoeostatic and signaling system involves relatively few molecular elements (Ca^2+^-permeable ion channels, Ca^2+^ pumps, Ca^2+^ solute carrier transporters, and Ca^2+^ buffers) which, by operating in concert, shape Ca^2+^ signals in the cytosol and in the organelles while at the same time preventing life-endangering Ca^2+^ overloads. Changes in [Ca^2+^]_i_ are sensed by numerous Ca^2+^-binding proteins, which translate Ca^2+^ signals into cellular activity.

**Figure 1.**
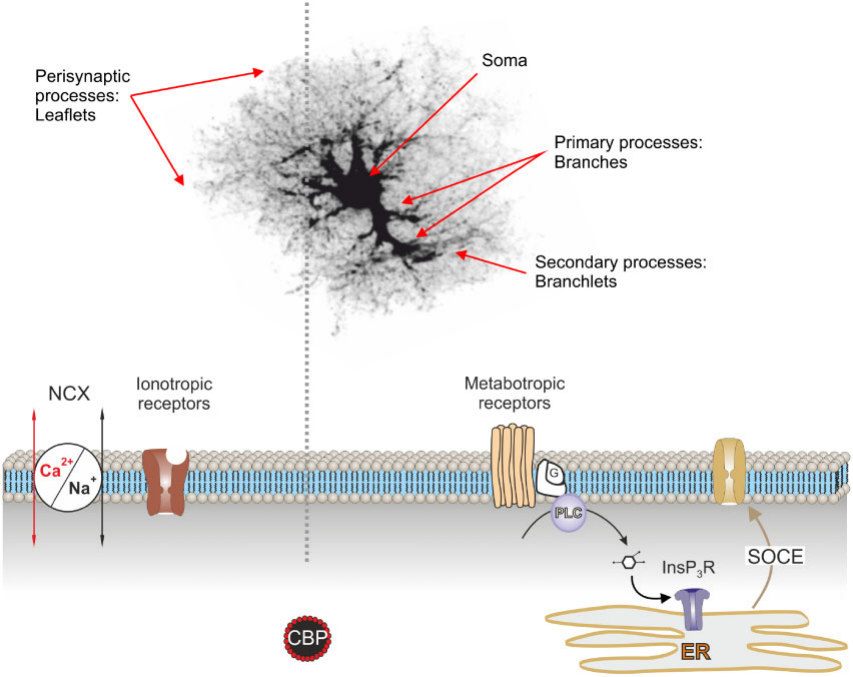
Morphofunctional Organization of Ca^2+^ Signaling Compartments in Protoplasmic Astrocyte. Morphological compartments of protoplasmic astrocyte^66^^,^^67^ are represented by (1) soma; (2) main processes also known as branches; (3) secondary to tertiary processes designated as branchlets; (4) peripheral parenchymal and perisynaptic processes known as leaflets; and (5) perivascular processes, which terminate with end feet plastering blood vessels; these latter are not shown on the figure. Calcium signaling in soma, branches, and branchlets are mainly associated with Ca^2+^ release from the ER with subsequent SOCE. This Ca^2+^ release is mediated by InsP_3_ receptors (InsP_3_R); InsP_3_ is synthesized by phospholipase C (PLC) linked to G-protein metabotropic receptors. Calcium signaling in the leaflets is associated with Ca^2+^ entry through ionotropic receptors (NMDA glutamate receptors or P2X purinoceptors) or Ca^2+^-permeable channels (such as, for example, TRPA1 channels). Plasmalemmal Ca^2+^ influx can also be mediated by the NCX operating in the reverse mode.

Astroglial Ca^2+^ signaling is characterized by a complex spatiotemporal organization, which reflects the elaborate astrocyte architecture. Furthermore, different types of astrocytes seemingly have distinct [Ca^2+^]_i_ dynamics with idiosyncratic underlying mechanisms. The morphological compartments of protoplasmic astrocytes (which are probably the most studied class of astroglia) are represented by (i) soma; (ii) main processes also known as branches; (iii) secondary to tertiary processes designated as branchlets; (iv) peripheral parenchymal and perisynaptic processes known as leaflets; and (v) perivascular processes, which terminate with end feet plastering blood vessels (Figure 1 ^66^^,^^67^). All these parts have distinct sizes (with soma being ∼10–15 µm, while primary processes ∼2–5 µm in diameter, an end feet size being in the 2–3 µm range, the branchlets having sub-micrometer diameters and leaflets representing structures with a thickness of ∼100 nm) and different organelle compositions. The perisynaptic leaflets are flat terminal processes with high surface-to-volume ratio and devoid of organelles.^68^ The terminal branchlets, however, may possess miniature mitochondria.^69^ These morphological arrangements are associated with distinct mechanisms of Ca^2+^ signal generation and distinct [Ca^2+^]_i_ dynamics in different astroglial compartments.

Numerous lines of evidence have demonstrated that Ca^2+^ signaling in distal processes develop independently from the soma and are often confined to leaflets or branchlets; these signals emerge as local micro- (or even nano-) domains of elevated [Ca^2+^]_i_. Focal [Ca^2+^]_i_ transients can either be spontaneous, with no association with neuronal activity,^70–73^ or local Ca^2+^ signals can result from neuronal activity and stimulation of astroglial receptors.^74–76^ As a rule, Ca^2+^ signals in the peripheral processes of protoplasmic astrocytes are shorter in duration than in the soma^77^^,^^78^ and are dominated (Figure 1) by plasmalemmal Ca^2+^ influx through Ca^2+^ permeable ionotropic receptors^55^^,^^79^ or transient receptor potential (TRP) channels^80^^,^^81^ or reversed Na^+^/Ca^2+^ exchanger (NCX).^82^^,^^83^ Calcium signals in the fine astrocytic branchlets appear more frequently than in the thicker branches; these local Ca^2+^ events in branchlets and branches can be amplified by Ca^2+^ released from the ER and mitochondria. The higher surface-to-volume ratio of branchlets allows larger plasma membrane Ca^2+^ influx and hence larger [Ca^2+^]_i_ fluctuations.^73^ As a result, local [Ca^2+^]_i_ fluctuations more frequently reach the threshold for Ca^2+^-induced Ca^2+^ release through InsP_3_ receptors. Hence, loss of fine astrocytic branchlets in pathological conditions such as epilepsy can be linked to reduced astrocytic Ca^2+^ activity.^84^

Somatic Ca^2+^ signals in protoplasmic astrocytes, as well as [Ca^2+^]_i_ transients in the primary processes are larger in amplitude and slower, are often synchronized with neighboring astrocytes (for example within the confines of a barrel in the somatosensory cortex^53^) and are originating from stimulation of metabotropic receptors and InsP_3_-induced Ca^2+^ release from the ER (Figure 1)^53^^,^^85^^,^^86^ that is associated with a consequent activation of store-operated Ca^2+^ entry (SOCE).^81^^,^^87^ Genetic deletion of the InsP_3_ receptor type II (the predominant astroglial InsP_3_ receptor) eliminates Ca^2+^ signals in the soma and in the primary processes, leaving [Ca^2+^]_i_ dynamics in branchlets and leaflets very much undisturbed or only partially suppressed.^88–91^

**Figure 2.**
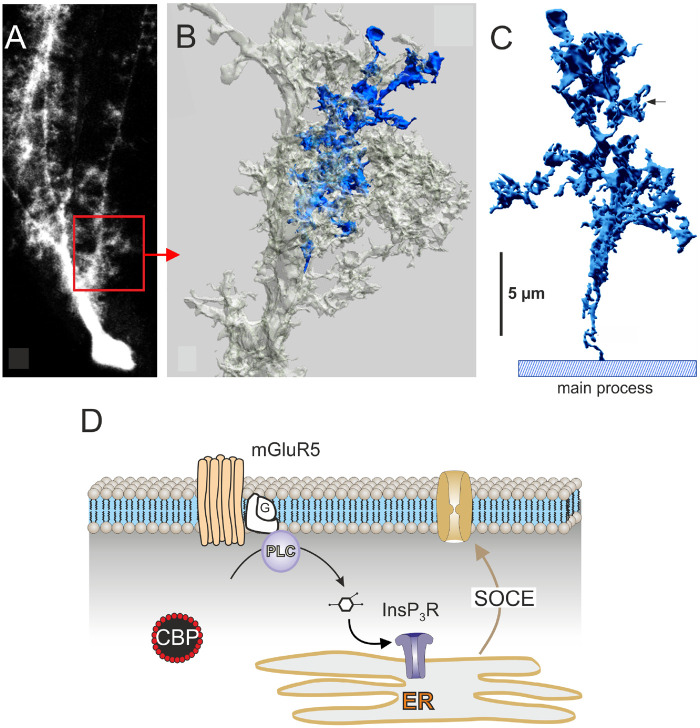
Formation of Ca^2+^ Microdomain in the Perisynaptic Appendages of Cerebellar Bergmann Glial Cells. Reconstruction of an appendage is based on electron microscopic data. (**A**) Fluorescence light micrograph of a dye-injected Bergmann glial cell is shown; the red square corresponds to the portion that was reconstructed from consecutive ultrathin sections. (**B**) One of the lateral appendages (marked in blue), arising directly from main process. (**C**) The same appendage is shown in isolation and at higher magnification. (**D**) Calcium signaling in the appendages of Bergmann glial cells is mediated solely through metabotropic receptors (mGluR5 or P2Y purinoceptors), which stimulate induced synthesis of InsP_3_ with subsequent InsP_3_-induced Ca^2+^ release from the ER and secondary SOCE. Modified from Ref. Grosche et al.^54^

This type of segregated Ca^2+^ signalling (ER-based Ca^2+^ release in soma and primary processes vs. plasmalemmal Ca^2+^ influx in leaflets) does not operate in all types of astrocytes. For example, in Bergmann glial cells (radial astrocytes in the cerebellum) Ca^2+^ signalling microdomains are associated with specific morphological structures—the appendages. These appendages emanate from the primary radially oriented processes of the Bergmann glial cells; each appendage contains mitochondria and projects leaflets that contact 50–70 synapses formed by axons of granular neurons (Figure 2A–C). Activation of parallel fibers triggers localized Ca^2+^ signals in these appendages; the [Ca^2+^]_i_ transients originate from activation of metabotropic receptors (mGluR5, P2Y purinoceptors, ET_B_ endothelin receptors, α_1_-adrenoceptors, and H_1_ histamine receptors) and InsP_3_ receptor-mediated Ca^2+^ release, with subsequent activation of SOCE.^54^^,^^92–95^ In neocortical astrocytes ryanodine receptor-mediated [Ca^2+^]_i_-induced Ca^2+^ release was shown to substantially contribute to α_1_-adrenoceptor-mediated Ca^2+^ signals^96^; conversely, this mechanism is absent in hippocampal astrocytes.^97^ Spontaneous [Ca^2+^]_i_ dynamics in the peripheral fine branchlets of cortical mouse astrocytes (examined in culture, in slices, and *in vivo*) were reported to originate from mitochondrial Ca^2+^ release through the flickering mitochondrial permeability transition pore.^98^ Local Ca^2+^ signals in branchlets (which possess ER) may involve combination of InsP_3_-induced Ca^2+^ release and plasmalemmal Ca^2+^ entry. Spatial restriction of [Ca^2+^]_i_ increases could result from local mitochondria, which act as powerful Ca^2+^ buffers^99^ and can localize [Ca^2+^]_i_ increases in astroglial processes.^75^ Another mechanism for functional compartmentalization of Ca^2+^ signals can be associated with plasmalemma-ER junctions that have been described in cultured primary astrocytes; these junctions are rich in InsP_3_ receptors, SERCA pumps, and NCX being thus a substrate for focal Ca^2+^ signaling.^100^ These examples form the mass of evidence demonstrating the diversity of astroglial Ca^2+^ signaling, which most likely changes depending on the physiological context, astrocyte morphology, age, and environmental settings.

Data on astroglial [Ca^2+^]_i_ dynamics *in vivo*, in awake animals, remain rather limited. It seems that sensory stimulation triggers large and global (ie, pan-cellular) [Ca^2+^]_i_ elevations controlled mainly by noradrenergic stimulation of α1 adrenoceptors.^53^^,^^65^^,^^101^ This cascade underlies a pan-cortical massive and spreading astroglial [Ca^2+^]_i_ increase in response to transcranial direct current stimulation.^102^ Arousal, attention, and vigilant state trigger global and widespread astroglial Ca^2+^ signals evoked by acetylcholine release from projections of the nucleus basalis of Meynert; these [Ca^2+^]_i_ responses are mediated through muscarinic ACh receptors and involve InsP_3_-induced Ca^2+^ release.^103^^,^^104^ Whether astrocytes in the *in vivo* setting communicate through propagating Ca^2+^ waves, which were characterized in detail *in vitro* and in brain slices,^32^^,^^105^ remains to be determined.

In summary, astrocytes possess a complex and spatially diverse Ca^2+^ signalling machinery that relies on several Ca^2+^ mobilizing pathways associated with ER Ca^2+^ release (mainly InsP_3_ receptor type II) and plasmalemmal Ca^2+^ entry through channels and the reversed NCX. Deciphering the targets for the Ca^2+^ signals in physiological and pathological contexts remains a pressing task. The remarkable heterogeneity of astroglial Ca^2+^ signaling is most likely linked to the extensive adaptive potential of astrocytes, which may tailor Ca^2+^ signalling toolkits to meet a multitude of challenges.

How Ca^2+^ signals translate into astroglial functional responses and how to find the physiological targets of [Ca^2+^]_i_ fluctuations remain largely unanswered questions. Similarly to other cells, astroglial Ca^2+^ signals regulate gene expression and provide for excitation-metabolic coupling; formation of [Ca^2+^]_i_ microdomains in astroglial branchlets immobilize mitochondria thus securing local metabolic support^106^ and Ca^2+^ signals may trigger astrocyte morphological plasticity.^58^^,^^59^ Astroglial Ca^2+^ signalling is implicated in secretion, both exocytotic and nonvesicular.^107^^,^^108^ Astrocytes are coupled to the regulation of functional hyperaemia^109^^,^^110^ through releasing vasodilators and vasoconstrictors, the secretion of which was initially linked to Ca^2+^ signals.^46^^,^^47^ Subsequent experiments however questioned this paradigm by demonstrating that suppression of astroglial InsP_3_-mediated Ca^2+^ signalling does not affect increases in local blood flow in response to sensory stimulation.^111–113^ Subsequently, astrocyte-vascular coupling was linked to extra-fast [Ca^2+^]_i_ transients occurring in the end feet shortly before vasodilatation.^77^^,^^78^ At the same time, the astrocytes were proposed to provide Ca^2+^-dependent slow tonic regulation of the vascular tone through continuous release of prostaglandins.^114^ Astroglial Ca^2+^ signals were also reported to undergo changes in sleep, while suppression of astroglial [Ca^2+^]_i_ dynamics by knocking out the InsP_3_ receptor type II affects slow-wave sleep with an increase in the number of micro-arousals, abnormal brain rhythms, and an increased frequency of slow-wave sleep state transitions and sleep spindles.^115^

In a pathological context, Ca^2+^ signaling controls reactive astrogliosis, the archetypal astrocytic defensive program. Challenge of astrocytes with damage-associated molecular patterns, such as ATP or β-amyloid triggers [Ca^2+^]_i_ rises,^116–119^ which initiate astroglial reactivity. Inhibition of astrocyte Ca^2+^ signaling by genetic ablation of InsP_3_ receptors^120^ or by pharmacological agents^121^ inhibits reactive astrogliosis.

### Astroglial Sodium Signaling

Changes in [Na^+^]_i_ are of particular significance for astrocytic homeostatic function, because the absolute majority of plasmalemmal transporters engaged in maintaining various aspects of molecular homeostasis are driven by transmembrane Na^+^ gradient. These transporters not only utilize Na^+^ gradients; their operation produces Na^+^ fluxes thus transporters being simultaneously the sensors and modifiers of [Na^+^]_i_. The resting [Na^+^]_i_ in astrocytes lies in the range of 15–20 mM,^81^^,^^122^^,^^123^ which is about twice that of neurons. Stimulation of astrocytes by neurotransmitters or by neuronal activity triggers substantial (up to 10–20 mM) increases in [Na^+^]_i_, which may last for tens of seconds.^56^^,^^82^^,^^122^^,^^124^^,^^125^ These [Na^+^]_i_ transients were shown to spread in the form of propagating [Na^+^]_i_ waves through individual cells (from processes to soma^125^) and into adjacent cells through gap junctions, thus creating intercellular [Na^+^]_i_ waves.^126^^,^^127^ In appearance therefore astroglial [Na^+^]_i_ dynamics is quite similar to [Ca^2+^]_i_ changes. The presence of complex [Na^+^]_i_ fluctuations together with the existence of numerous Na^+^-dependent molecules (or Na^+^-sensors) led to the concept of astroglial Na^+^ signaling.^128^^,^^129^

**Figure 3.**
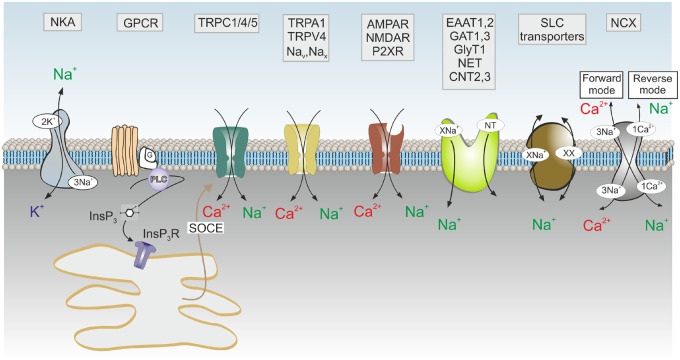
Membrane Molecular Pathways of Na^+^ Signaling in Astrocytes. Influx of Na^+^ occurs though (1) Na^+^-permeable channels, which include ionotropic receptors (AMPAR, NMDAR, P2XR - AMPA, NMDA glutamate receptors and ionotropic purinoceptors); channels of the TRP family (TRPC1/4/5 channels that operate as a part of store-operated Ca^2+^ entry and hence generate Na^+^ influx in response to the depletion of ER Ca^2+^ stores; as well as TRPA and TRPV channels); voltage-dependent Na_v_ channels and [Na^+^]_o_-activated Na_x_ channels; (2) through Na^+^-dependent SLC transporters that include excitatory amino acid transporters EAAT1,2, GABA transporters GAT 1,3, glycine transporters GlyT, NA transporters NET or concentrative adenosine transporters CNT2/3. The main pathway for Na^+^ exit is provided by Na^+^-K^+^ pump, NKA. The Na^+^-Ca^2+^ exchanger NCX fluctuates between forward and reverse mode and couples Na^+^ and Ca^2+^ signaling. Modified from Verkhratsky et al.^130^

Despite overt similarity between [Ca^2+^]_i_ and [Na^+^]_i_ dynamics in astrocytes, the underlying mechanisms are quite distinct. Astrocytic Na^+^ signals rely on plasmalemmal Na^+^ movements only, as no Na^+^ storing structures exist in the cells (Figure 3). Plasmalemmal Na^+^ entry is mediated by cationic channels, which include ionotropic receptors and TRP channels. All these channels have considerable Na^+^ permeability; the pCa/pNa for P_2_X and NMDA receptors and TRP channels varies between 2 and >5, but given the high Na^+^ concentration in the interstitial fluids, Na^+^ fluxes through these channels are predominant.^55^^,^^81^ Astrocytes in the subfornical organ possess a specific Na^+^ channel, classified as Na_x_ channels (which were initially cloned from astrocytes^131^) that are activated by increases in extracellular Na^+^ above 140 mM. These channels allow subfornical astrocytes to monitor blood Na^+^ concentration and contribute to systemic regulation of Na^+^ homeostasis.^132^ Expression of voltage-gated Na_v_1.2, Na_v_1.3, Na_v_1.5, and Na_v_1.6 channels in astrocytes has been detected at both mRNA and protein levels; however, their functional relevance remains to be tested.^133^

The second route for Na^+^ entry is associated with Na^+^-dependent transporters of which Na^+^-dependent neurotransmitter transporters contribute the most. These include excitatory amino acid transporters types 1 and 2 (EAAT1/SLC1A3 and EAAT2/SLC1A2^134^^,^^135^); GABA transporters type 1 and 3 (GAT-3/SLC6A1 and GAT-3/SLC6A12^136^); glycine transporters type 1 (GlyT1/SLC6A9^137^); NA and dopamine transporters (NET/SLC6A2 and DAT/SLC6A3^138^) and Na^+^-coupled neutral amino acid transporters SNAT3/SLC38A3 and SNAT5/SLC38A5.^139^ All these transporters are of paramount importance for neurotransmitter homeostasis and neurotransmission maintenance. In addition, Na^+^ fluxes are created by homeostatic transporters such as Na^+^-K^+^-Cl^−^ co-transporter NKCC1/SLC12A2, Na^+^-dependent d-glucose transporter SGLT1/SLC5A1 or Na^+^-dependent vitamin C transporter SVCT2/SLC23A2.^140–142^

Extrusion of Na+ from astrocytes is mediated solely by Na+/K+ ATPase (NKA). Inhibition of NKA in cultured astroglia by ouabain or by removal of extracellular K+ results in an increase in [Na+]_i_ up to 30–40 mM within ∼5 min.^122^^,^^143^^,^^144^ This reveals a substantial basal Na^+^ influx into astrocytes mediated probably by all types of Na^+^ permeable channels and possibly transporters such as Na^+^-H^+^ exchanger (NHE) or Na^+^-dependent bicarbonate symporter (NBCe1). The NKA in astrocytes incorporates the α2 subunit; which is not expressed in neurons (which possess α 1 and α 3 subunits). As a result, the affinity of astroglial NKA to K^+^ is substantially lower than in neurons. The EC_50_ for K^+^ for astroglial NKA composed from α2/β1 subunits is ∼3.6 mM, while EC_50_ for K^+^ in neuronal NKA (formed by α1/β1, α1/β2, α3/β1, or α3/β2 subunits) varies between 0.25 and 0.65 mM.^140^ Thus, differences in structure determine NKA function: at physiological levels of interstitial [K^+^] (∼3–3.5 mM) the neuronal NKA K^+^ binding sites are fully saturated; whereas for astrocytic NKA half of the K^+^ binding sites remain unoccupied. Consequently, an increase in interstitial [K^+^] activates astroglial NKA, which is the main mechanism for extracellular K^+^ sensing and buffering. Neuronal NKA is activated solely by an increase in [Na^+^]_i_. The NKA-dependent transport of K^+^ and Na^+^ in astrocytes maintains ion gradients critical for operation of homoeostatic transporters; in essence, the NKA acts as the master regulator of astroglial homoeostatic physiology. Increases in NKA transport, which accompany neuronal activity (to buffer K^+^ or to expel excessive Na^+^ entering the cell in the course of glutamate uptake) are also linked to astroglial l-lactate production and hence are central for the operation of astrocyte-to-neurone-lactate shuttle (ANLS).^45^ Operation of astroglial NKA is regulated by β-adrenoceptors^145^ and possibly by endogenous ouabain-like molecules.^146^ Normal operation of astroglial NKA is needed for learning,^147^ whereas loss-of-function mutations in the α2 subunit is associated with familial hemiplegic migraine type 2.^148^^,^^149^

The second key player of Na^+^ signaling is represented by the NCX; astrocytes express all three subtypes of this exchanger (NCX1/SLC8A1, NCX2/SLC8A2, and NCX3/SLC8A3). These subtypes are quite similar from the functional point of view, exchanging Na^+^ and Ca^2+^ with a 3:1 stoichiometry.^150^^,^^151^ The reversal potential of the astrocytic NCX is quite close to the resting membrane potential and hence even minor changes in [Na^+^]_i_ or small depolarizations turn NCX into the reverse mode when it brings Ca^2+^ into the cell in exchange for Na^+^; in this mode, the NCX generates a [Ca^2+^]_i_ rise while accelerating recovery to resting [Na^+^]_i_.^83^^,^^152^^,^^153^ Conversely, when [Ca^2+^]_i_ rises the NCX is forced into the forward mode in which it assists the recovery of [Ca^2+^]_i_ transients while producing Na^+^ influx.^123^ Thus, the NCX acts as a central molecule linking Ca^2+^ and Na^+^ signaling.

Similar to [Ca^2+^]_i_ dynamics, astrocytic Na^+^ signals may be confined to microdomains. Such local subcellular [Na^+^]_i_ transients have been characterized in Bergmann glia and in hippocampal protoplasmic astrocytes.^57^^,^^124^ The molecular mechanisms behind such localizations remain unknown; apart from the plasmalemma localized channels and transporters, there is no evidence for cytosolic Na^+^ buffers/binding sites, which may account for the localization of [Na^+^]_i_ rises. The Na^+^ transporters in the plasmalemma endowed with Na^+^ binding sites may act as some sort of highly localized and relatively immobile Na^+^ buffers. Alternatively, Na^+^ (and other cations) may be trapped in tiny leaflets by binding sites associated with the inner side of the plasma membrane.^154^ Besides forming microdomains, Na^+^ signals can propagate from cell to cell by diffusion through gap junctions; the speed of these waves in [Na^+^]_i_ may reach 100–150 mm/s.^125^^,^^155^

There are surprisingly large varieties of Na^+^ sensors, which act as effectors of Na^+^ signals. The larger class of molecules governed by [Na^+^]_i_ is represented by the SLC membrane transporters, which fulfill an astroglial homoeostatic function. Changes in [Na^+^]_i_ may affect not only the efficacy of transports but also change their operational direction. The reversal is well documented for NCX (see above) and can also occur to some other transporters, such as, for example, GABA or glycine transporters, which have been shown to reverse in physiological settings following an increase in [Na^+^]_i_.^156–158^ Increases in [Na^+^]_i_ may translate to various biochemical and cellular responses though action on enzymes; in an astroglial context Na^+^ regulates glutamine synthetase thus affecting availability of glutamine for the glutamate (GABA)-glutamine shuttle.^159^ Cytoplasmic Na^+^ ions are also known to modulate or open various types of ion channels, such as, for example, Na^+^-dependent K^+^ channels or K_ir_4.1 inward rectifying K^+^ channels.^160^^,^^161^ Nonetheless in astrocytes the SLC transporters remain the main target; astroglial Na^+^ signaling therefore was proposed as a mechanism for rapid tuning of astroglial homeostatic cascades to neuronal activity.^128^

### Other Ions in Astroglial Excitability

#### Chloride

Chloride, the major inorganic anion in the living tissues, is a likely contributor to astroglial ionic excitability. There are multiple indications for signaling role of intracellular Cl^−^. Changes in [Cl^−^]_i_ regulate plasmalemmal channels (for instance *Slo*-2 K^+^ channels^160^ or TRPM7 channels^162^) and transporters (such as Na^+^/${\text{HCO}}_{3}^{-}$ transporter NBCe1-B^163^ or Na^+^/H^+^ exchanger HNE^164^); furthermore [Cl^−^]_i_ affects the activity of G proteins.^165^^,^^166^ Another signaling cascade directly regulated by [Cl^−^]i is associated with WNK (With No lysine [K]) serine/threonine protein kinases.^167^^,^^168^ Finally, dynamic changes in [Cl^−^]_i_ contribute to the regulation of several fundamental cellular processes such as cell differentiation and death.^169^^,^^170^

At the same time, Cl^−^ is the central ion for mediating inhibitory currents in neural cells, and hence fluctuations in [Cl^−^] in the interstitial fluid are of paramount importance for balancing neurotransmission. Experiments on cultured astrocytes demonstrated that astrocytes maintain high [Cl^−^]_i_ ranging between 20 and 50 mM, which corresponds to E_Cl_ = −35 mV.^171–173^ These data have not been universally confirmed in experiments *in situ* in acute brain slices. In Bergmann glial cells of the cerebellum Cl^−^ imaging indeed revealed high [Cl^−^]_i_ of around 50 mM in newborn and 35 mM in mature mice.^174^ In contrast probing astrocytes in acute hippocampal slices with gramicidine-based perforated patch-clamp estimated much lower [Cl^−^]_i_ at 3–4 mM.^175^ Certainly mapping astroglial [Cl^−^]_i_ *in vivo* is of pressing importance; as it may reveal either regional or state-dependent differences.

Astrocytic Cl^−^ homeostasis depends on Cl^−^ diffusion through several sets of anion channels that include (i) GABA_A_ and glycine receptors; (ii) inwardly rectifying chloride channels ClC-1, -2, and -3; (iii) Ca^2+^-dependent Cl^−^ channels; (iv) anion channels of the Bestrophin (*Best*) family and by (v) volume-regulated anion channels VRAC or SWELL1.^176–180^ All these channels mediate Cl^−^ efflux or influx depending on the [Cl^−^]_i_; at the same time molecular mechanism(s) for Cl^−^ accumulation into astrocytes remains to be identified. The only known Cl^−^ accumulating transporter, Na^+^/K^+^/Cl^−^ co-transporter NKCC1/SLC12A1, has been frequently identified in astrocytes in culture, however, whether NKCC1 operates *in situ* or *in vivo* remains controversial.^140^^,^^181^ Operation of several other transporters (such as GABA transporters and EAATs) is also associated with Cl^−^ fluxes.^174^^,^^182^ The sensors for Cl^−^ signaling in astrocytes are yet to be fully characterized; the role of astrocytes as a source for Cl^−^ to maintain inhibitory transmission has been proposed^172^ and demonstrated in hippocampal slices.^183^

#### Potassium

Life on Earth is believed to have emerged around four billion years ago in a Na^+^-rich Primordial Ocean. Surprisingly, the cytoplasm of most cells has high K^+^ and low Na^+^ concentrations. Several hypotheses explaining this phenomenon have been developed. For example, protocells could have emerged in the K^+^ enriched vents at the bottom of the ocean; or they may have appeared in the inland basins molded from K^+^ rich clay and filled with rainwater.^184^ Be this as it may, K^+^ plays a vital role in cellular life. High [K^+^]_i_ is required for protein synthesis and sets the cell membrane potential, while K^+^ efflux repolarizes the cell membrane following action potentials, excitatory postsynaptic potentials, and dendritic spikes in neurons.

Hence, neuronal activity is associated with substantial K^+^ fluxes across astroglial membranes. Astrocytes remove excess K^+^ at the peak of neuronal activity and then return K^+^ back to restore neuronal ionic gradients; in pathology, astrocytes are capable of redistributing K^+^ through the syncytial networks. Notably, most of the K^+^ removed by astrocytes from the synaptic cleft during neuronal activity comes from K^+^ efflux through ionotropic glutamate receptors, predominantly of NMDA type.^185–187^ Accumulation of K^+^ into astrocytes is mainly mediated by NKA (discussed in the previous section), while K^+^ efflux is mediated by inwardly rectifying K^+^ channels.^140^ This scenario implies emergence of short-lived K^+^ microdomains in perisynaptic astroglial processes, but whether these domains exist remains to be experimentally seen. The mechanisms of formation of K^+^ microdomains are similarly unknown. Recently, the role of intramembrane negative charges preventing K^+^ diffusion^154^ has been suggested. Whether dynamic changes in astroglial [K^+^]_i_ have a signaling role and directly modulate cellular functions similarly needs to be tested. One testable possibility is that K^+^-mediated depolarization can affect voltage-dependent steps of glutamate transporter cycle, hence, affecting glutamate uptake.^188^

#### Protons

Neuronal activity is accompanied by a transient decrease in astroglial [H^+^]_i_. This phenomenon is known as “depolarization-induced alkalinization” and results in accumulation of H^+^ in the extracellular space.^189^ Astroglial alkalinization is linked to activation of the Na^+^/${\text{HCO}}_{3}^{-}$ transporter NBCe1/SLC4A4. This transporter contributes to regulation of astroglial metabolism through stimulation of cAMP production and subsequent increase in glycolysis.^190^ Another metabolic pathway controlled by H^+^ is represented by phosphofructokinase; activation of the latter is perceived as a key step in stimulation of astrocyte-neuronal lactate shuttle.^191^

## Astroglial cAMP Excitability

The discovery of the first second messenger cAMP is linked to the studies of glycogen regulation. Under the mentorship of Carl Ferdinand Cori, who won a Nobel Prize in 1947 for identifying the mechanism of glycogen metabolism, Earl Wilbur Sutherland revealed that the action of adrenaline on glycogen degradation is mediated by cAMP.^192^^,^^193^ For this discovery, Sutherland was awarded the 1971 Nobel Prize in Physiology or Medicine. Unlike the technology for measuring cellular Ca^2+^, which emerged from two chemical inventions: a new family of calcium chelators with high affinity for Ca^2+194^and a method for trapping such substances inside intact cells by means of nonpolar ester derivatives,^194^ the methods to measure [cAMP]_i_ at cellular level appeared much later. The cAMP indicators are based on the fluorescence resonance energy transfer (FRET), a quantum-mechanical, nonradiant, transfer of energy from the excited state of a donor fluorophore to the ground state of a neighboring acceptor chromophore or fluorophore. The acceptor must absorb light at roughly the same wavelengths as the donor emits and if the donor and acceptor are located within <10 nm distance from each other, FRET may occur.^195^ Although the very first cAMP FRET sensors were available already in 1991,^196^ their usage was hindered by the need to inject FRET holoprotein nanosensors into individual cells, which prevented a wider application. The problem was solved by utilizing the green fluorescent proteins (GFPs) from jellyfish, engineering smaller FRET constructs which are introduced into cells via plasmid transfection. Cyclic AMP exerts its cytoplasmic effects via cAMP-binding proteins including cAMP-dependent protein kinase (PKA), cAMP-gated ion channels, and isoforms of exchange protein directly activated by cAMP (Epac). Full length proteins or only cAMP-binding domains of these target proteins, for example using Epac, together with variants of GFPs, were used to make the FRET nanosensors.^197–199^

Given the relatively complex design of cAMP nanosensor, it is not surprising that the first single-cell measurements of [cAMP]_i_ in astrocytes emerged only recently.^200^ In these experiments the expression of the FRET-based cAMP sensor, Epac1-camps, utilizing a single chain cAMP binding domain of the Epac1 protein,^198^ revealed a uniform distribution of the nanosensor fluorescence throughout the cytosol, but was excluded from the nucleus, indicating that [cAMP]_i_ may be homogeneously distributed at rest in the cytoplasm, yielding levels from 0.1 to several µM of [cAMP]_i_.^201^ While there is evidence that in microglia cAMP may accumulate at cell processes,^202^ this needs to be further addressed in astrocytes.

Stimulation of astrocytes with adrenaline at 29 nM induced a half-maximal increase in [cAMP]_i_, consistent with the action of β-adrenergic receptors.^200^ The increase in [cAMP]_i_ was characterized by a monoexponential rise to a plateau with a time-constant of ∼15 s, much slower than the agonist-induced increases in [Ca^2+^]_i_ in astrocytes.^203–205^ The steady-state level of [cAMP]_i_ represents the balance between the production of cAMP by adenylyl cyclases (AC) and its enzymatic degradation by phosphodiesterases.^206^ Unlike in other cells, where oscillations in [cAMP]_i_ were recorded and were considered to be due to an interaction with Ca^2+^ signaling,^207^ measurements in astrocytes failed to detect such oscillations.^208^

**Figure 4.**
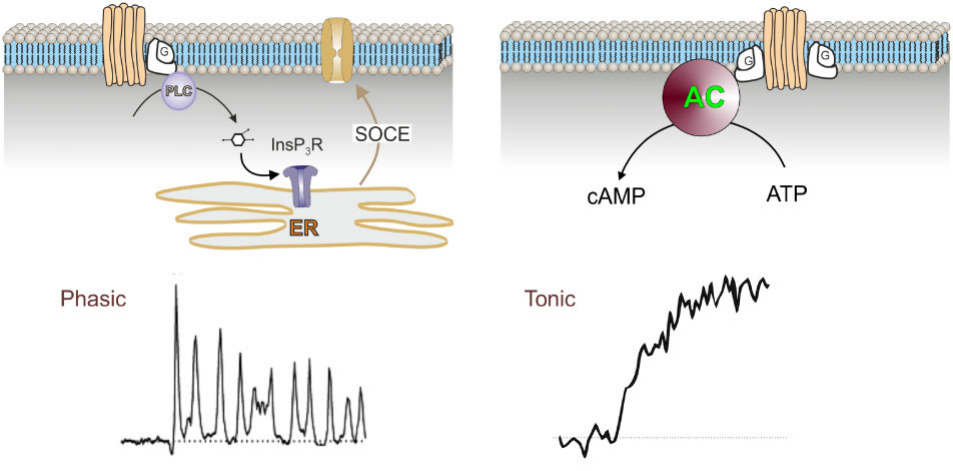
Distinct Temporal Dynamics of cAMP and Ca^2+^-Excitability in Astrocytes. Activation of astrocytic metabotropic receptors coupled to Gq proteins leads to phasic oscillations in intracellular Ca^2+^ levels (left), while the activation of metabotropic receptors coupled to Gs proteins leads to tonic long-lasting increase in cAMP-dependent PKA activity without oscillations (right). Cyclic AMP is produced by AC from ATP. PLC, phospholipase C; InsP_3_R, receptor. Modified from Horvat et al.^202^

However, despite the fact that cAMP and Ca^2+^ signaling operate in different time domains in astrocytes, there is an interaction between these pathways.^203^ Both pathways are activated by G-protein coupled receptors. While the elevation in [cAMP]_i_ is tonic, lasting several minutes, the swift changes in [Ca^2+^]_i_ are phasic, often exhibiting oscillations (Figure 4 ^203^^,^^208^). This dichotomy in kinetics of Ca^2+^ and cAMP signals was recently confirmed also *in vivo*,^65^ demonstrating that the two signaling mechanisms drive downstream cellular processes with distinct temporal characteristics.

The cross-talk between the cAMP- and Ca^2+^-signaling in astrocytes, reflects a mode of optimization of cellular responses upon receptor activation. The molecular mechanisms underlying the cross-talk between the Ca^2+^ and cAMP responses in astrocytes in health and disease remain to be studied. However, as observed in other cell types, Ca^2+^ may modulate the activity of the ACs and PDEs, through calmodulin, while cAMP-dependent signaling may affect Ca^2+^ transport mechanisms and may regulate gene expression via cAMP/PKA, therefore affecting the production of proteins of the Ca^2+^ signaling cascades. Moreover, Ca^2+^ oscillation frequency appears to determine gene transcription,^209^^,^^210^ thus the cAMP-mediated regulation of Ca^2+^ oscillations may alter astroglial gene expression.

Astroglial glycogen represents an energy reserve, which is used during increased activity to support many CNS functions, including memory formation and consolidation.^211^ When astrocytes are stimulated, for example by NA,^212^ this results in an increased glucose uptake, glycogenolysis, and glycolysis with l-lactate as the end glycolytic product despite the normal oxygen levels (ie, aerobic glycolysis, known also as the Warburg effect). Glycogen-derived l-lactate exits astrocytes through monocarboxylate transporters (MCTs) 1 and 4 and/or yet unidentified ion channels^213^ to enter neurons through the MCT2, where it is used in oxidative metabolism (ie, astrocyte-neurone-lactate-shuttle hypothesis^45^). Moreover, l-lactate can also act as an extracellular signal where it binds to l-lactate metabotropic receptors or to yet unknown receptors.^214–216^

Aerobic glycolysis together with glycogenolysis is regulated in astrocytes by a variety of receptors on the surface of astrocytes that are linked to intracellular Ca^2+^- and/or cAMP-pathways, such as ARs and purinoreceptors. Upon stimulation of LC neurons, NA is released, with subsequent activation of metabotropic adrenoceptors and increases in astrocytic [Ca^2+^]_i_ and [cAMP]_i_.^53^^,^^65^ The contribution of Ca^2+^ and cAMP as second messengers to the regulation of aerobic glycolysis and glycogenolysis in astrocytes remains unclear and even controversial. It is thought that aerobic glycolysis and glycogenolysis are primarily elevated through the cAMP-dependent pathway in astrocytes,^217^^,^^218^ although there is evidence that Ca^2+^ signals might also be involved.^219^

In conclusion, astroglial noradrenergic signaling, involving Ca^2+^ and cAMP regulates many cellular processes affecting the function of astrocytes and neighboring neurons in health and disease. This intracellular excitability provides regulatory clues in distinct space and time domains, which underlies the capacity of adapting to dynamic and life-long changes that occur during the function of the CNS in health and disease.

## Recapitulation

Astrocytes are an indispensable part of the nervous tissue, which together with neurons and other neural cells produce a cellular fabric responsible for brain function. Homoeostatic cascades in the astrocytes, which support the most fundamental functional properties of the CNS, are tightly correlated with neuronal activity and tissue demands. This coordination is a function of astroglial excitability mediated through spatiotemporal fluctuations of intracellular ions and second messengers.

## Funding

A.S. work was supported by Russian Science Foundation (grant number 20-14-00241); R.Z. is supported by grants from the Slovenian Research Agency (P3 310, J36790, J3 9266, J3 7605), CIPKEBIP, COST Nanonet, COST Mouse Ageing, and COST CM1207 – GLISTEN.

## Conflict of Interest Statement

None declared.
